# Amniotic Fluid Lactate Level as a Diagnostic Tool for Prolonged Labour

**DOI:** 10.34763/jmotherandchild.20202403.2027.d-20-00011

**Published:** 2020-12-03

**Authors:** Kinga Pospiech, Krzysztof Czajkowski

**Affiliations:** 1 2nd Department of Obstetrics and Gynecology, Medical University of Warsaw, Warsaw, Poland

**Keywords:** amniotic fluid lactate, caesarean section, prolonged labour, dysfunctional labour, arrested labour, vaginal delivery

## Abstract

Prolonged labour can lead to postpartum complications and adverse outcomes for both mother and baby. Measurable parameters can help in the active management of labour, timely diagnosis of dystocia and in the choice of the method of delivery. Progressive uterine contractions are necessary to complete labour successfully. Myometrial fatigue during prolonged labour causes a change from aerobic to anaerobic metabolism, resulting in an accumulation of intramuscular lactic acid and probably a subsequent increase in amniotic fluid lactate concentration. High amniotic fluid lactate level has been associated with ineffective uterine contractions leading to labour arrest. A considerable number of studies conducted so far indicate that the level of lactate in amniotic fluid may be a new non-invasive diagnostic tool for early prediction of prolonged labour and the need for immediate obstetric intervention. Low amniotic fluid lactate level may facilitate a decision to continue vaginal labour by oxytocin augmentation. A high level of amniotic fluid lactate is associated with surgical obstetric procedures. Measuring amniotic fluid lactate level might simplify the patient’s allocation to a group, which will benefit from the administration of oxytocin and to a group that will not benefit from further prolongation of labour. This study aimed to briefly review current knowledge on amniotic fluid lactate concentrations measured using standard biochemical methods during the first stage of labour following normal pregnancy, as a possible diagnostic tool for prolonged labour. For this purpose, PubMed, EMBASE, Medline (1990 to July 2020) trials register and reference lists of relevant articles were searched.

## Introduction

Monitoring the progress of labour continues to be one of the main obstetric problems worldwide. It is estimated that about 20% of all deliveries are complicated by abnormal labour progression (1) (other terms used interchangeably: labour dystocia, labour disorders, dysfunctional labour, prolonged labour and arrested labour). It is frequently associated with an increased risk of instrumental or surgical interventions, low Apgar score at 5 min, admission to neonatal intensive care unit, grade III-IV perineal tear, above-average maternal blood loss (2), higher risk of potential post-partum infections and a prolonged maternal and newborn hospitalization (3).

Considering the above, a failure to progress causes an increase in numerous delivery complications, so a better understanding of the risk factors of dysfunctional labour progression may help in the timely diagnosis of dystocia, active management of labour and ultimately help in choosing the best method of delivery for a particular patient.

Maternal risk factors of prolonged labour include advanced maternal age (4, 5, 6), high body mass index (BMI), excessive weight gain during pregnancy, and high maternal pre-pregnancy BMI (7, 8). Large estimated infant birthweight, large head circumference, occiput posterior position are foetal risk factors (3, 9, 10).

The assessment of the progress of labour should be based on vaginal examination. It is estimated by measuring the changes in cervical effacement and dilatation during the first stage of labour and subsequently assessing foetal descent during the second stage. However, no precise definition of the diagnosis of dystocia has been presented to date.

This study aimed to briefly review the current knowledge on amniotic fluid lactate concentrations measured using standard biochemical methods during the first stage of labour following normal pregnancy, as a possible diagnostic tool for prolonged labour. A literature search for relevant articles was conducted using PubMed, EMBASE, Medline databases from 1990 to July 2020.

## Physiology of Myometrial Contractions

Regular uterine contractions of progressively increasing frequency and duration are necessary to complete labour successfully. During each uterine contraction, poorly perfused tissues undergo anaerobic metabolism. In these anaerobic conditions, pyruvate generated by glycolysis is reduced to lactate in a reaction catalysed by lactate dehydrogenase (LDH) in the presence of nicotinamide adenine dinucleotides (NADH) as a cofactor, yielding merely 2 adenosine triphosphate (ATP). It is worth noting that anaerobic glycolysis is significantly less efficient at using the energy from glucose: only 2 ATP are produced per glucose molecule, compared to *38* ATP per glucose molecule nominally produced by aerobic respiration. High lactate level is associated with a decrease in pH to 6.9–6.4 as well as a reduction in phosphofructokinase activity—one of the most important regulatory enzymes of glycolysis and inhibitors of glycolysis and glycogenolysis (1, 11, 12). The decrease in pH affects membrane currents, including decreased entry of Ca2?, leading to reduced contractions resulting in labour dystocia (13, 14). After a uterine contraction, lactate and the remaining products of anaerobic metabolism are removed and the circulatory system supplies oxygenated blood to the myometrium. This process ensures that lactate values and effective uterine contractions are maintained at the desired level. Conversely, during inadequate myometrial contractions, there is a decrease in the strength of uterine contractions as lactate and other products accumulate in the tissue, leading to a failure to progress (15).

The studies conducted to date indicate that the anaerobic pathway of myometrium glucose metabolism results in a high lactate level, which, in turn, is expected to increase the presence of lactate in amniotic fluid. In 2004, Quenby et al. compared pH, blood gas and lactate level in blood samples taken from uterine low segments in women undergoing an emergency caesarean section due to dysfunctional labour, an emergency caesarean section due to other indications during labour or an elective caesarean section. The study showed significantly lower pH and pO2 levels as well as a high level of lactate in myometrial blood samples from women with dysfunctional labour as compared to other groups (15). Recent studies have shown that myocytes produce and transport lactic acid to extracellular space. In 2009, Akerud et al. detected monocarboxylate transporters (MCTs: MCT-1 and MCT-4) in biopsies from the uterus. MCTs transport molecules with one carboxylate group, such as lactate and pyruvate, across biological membranes. In the case of myometrial cells and amniotic fluid, they transport molecules across two layers of placental membranes. The same type of transporters is present in skeletal muscle cells and other tissues (16).

## Amniotic Fluid Lactate (AFL) Level as a Diagnostic Tool

In the past, there were numerous attempts at using medical imaging techniques to assess the progress of labour. However, no significant changes have been made to improve the assessment of the course of labour since 1950, when Friedman presented a partograph as a tool providing accurate information on the progress of delivery (besides a few modifications made by the WHO in 1990). A partograph is currently recommended by the WHO in standard labour management and care.

The following observations are recorded on a partograph: maternal condition including blood pressure, heart rate, temperature, total amount of administered fluids, the amount of administered oxytocin and other drugs, foetal heart rate, a precise time of the rupture of the membranes and the colour of the drained amniotic fluid, the presence of meconium and blood, frequency, duration and strength of uterine contractions, as well as descent of foetal head on abdominal palpation. During the first stage of labour, the vaginal examination should be performed every 4 h to minimise the risk of infection. Cervical dilatation should be recorded on a partograph from the beginning of the active stage of labour (at least 4 cm dilatation). It progresses typically at a rate of 1 cm per hour or more (17).

The effectiveness of a partograph in monitoring the progress of labour is still debated. A Cochrane review from 2013 analysed six studies and 7706 female patients to assess the differences between the group which used a partograph during labour and the group that did not use it. The data did not show a statistically significant difference between the two groups in terms of the percentage of caesarean sections, instrumental vaginal delivery or Apgar score less than seven at 5 min (18). In a trial of Wiberg-Itzel et al. from 2016, labour dystocia was diagnosed in about 50% of 2793 deliveries based on partograph recordings, however after the administration of oxytocin over 64% of cases concluded with a spontaneous vaginal delivery (19). There are a number of studies published between 2008 and 2017, which explored the possibility of measuring AFL concentration to assess the unsatisfactory progress of labour and the need for a caesarean section. A prospective observational study conducted by Wiberg-Itzel et al. in 2008 eventually enrolled 54 women who met the inclusion criteria, had volunteered to have an intrauterine pressure (IUP) catheter inserted and had a caesarean section or a vaginal instrumental delivery due to arrested labour (experimental group) or spontaneous vaginal delivery (control group). To measure the AFL level, the patients had at least two samples of amniotic fluid taken 60 min apart in the active phase of labour. The average amniotic fluid lactate concentration in the control group was 8.9 mmol/l (range 6.6–10.8), while the AFL level increased to an average value of 10.9 mmol/l (range 8.0–16.1) (p?0.001) in the group with an operative delivery due to labour dystocia. Moreover, 86% of women with a high AFL level ?10.1 mmol/l had a caesarean section or a vaginal instrumental delivery due to dystocia. The authors concluded that there was a strong association between a high AFL level ?10.1 mmol/l and arrested labour (20).

In 2010, the same researchers evaluated AFL concentration measurements of 825 women in the active stage of labour. The second trial confirmed the diagnostic value of a high AFL level ?10.1 mmol/l in predicting the need of a surgical delivery due to labour dystocia (adjusted OR, 5.4, 95% CI, 3.2–9.1), while an AFL level below ?10.1 mmol/l was associated with an increased chance of a spontaneous vaginal delivery (adjusted OR, 2.7, 95% CI, 1.7–4.8). The average time for the diagnosis of arrested labour was 19.4 h in the group of patients with the AFL level ?10.1 mmol/l as compared to 11.3 h in the group with the AFL level ?10.1 mmol/l (21).

In 2015, Murphy et al. analysed the data of 905 nulliparous women at term (?37 weeks) with a spontaneous onset of labour who had their AFL concentration measured during labour using a point-of-care device. The women were divided into three groups according to the AFL level (0-4.9 mmol/l, 5-9.9 mmol/l, ?10 mmol/l. The frequency of caesarean sections was 17.5% in the group with the highest AFL level ?10 mmol/l, which constituted a threefold and a fivefold increase as compared to the 5–9.9 mmol/l group (6.1%) and 0–4.9 mmol/l group (3.4%), respectively. A multivariable analysis demonstrated that an AFL concentration ?10mmol/l is an independent risk factor for a caesarean section (OR, 3.35; 95% CI, 1.73–6.46) (22).

In 2016, Wiberg-Itzel et al. conducted a prospective multicentre study assessing whether a high AFL level could be used as a predictor of an invasive delivery and labour outcomes before the administration of oxytocin during dysfunctional labour. For this purpose, the authors selected 638 amniotic fluid samples collected within 30 min before oxytocin augmentation (from 5418 samples). Unsatisfactory progress of labour was defined as cervical dilatation progress at a rate of less than 1 cm per hour or the arrest of labour for at least 2 h in the active stage of labour (?3cm cervical dilatation). It was shown that 69.4% of this augmented labour concluded with a spontaneous vaginal delivery, including 23.8% with active stage of labour prolonged to 12 h. A high AFL level ?10.1 mmol/l correlated with either an increased frequency of a surgical delivery (p?0.001) or a prolonged active phase of labour ?12 h (*p* ? 0.04). Furthermore, epidural anesthesia was administered significantly more often in patients with an AFL concentration ?10.1 mmol/l (*p* ? 0.007). Another significant observation was a higher frequency of complications, that is, postpartum fever (?38?C) (*p* ? 0.01) and postpartum hemorrhage ?1.5l (*p* ? 0.04). A high AFL level had a 4.5 times higher sensitivity in predicting caesarean section than a low level of AFL. Thus, measuring the value of AFL along with using a partograph allows health care professionals to monitor the progress of labour in an optimal way. It was shown that 84% of patients requiring a caesarean section could be identified based on of partograph recordings and a high level of AFL. Furthermore, 39% of patients who had caesarean section could have had caesarean section performed earlier, thus avoiding painful and prolonged labour (19).

In 2017, Wiberg-Itzel et al. published the results of a randomised controlled trial of a new treatment for labour dystocia based on the oral intake of bicarbonate (23), the strategy used in improving sports performance in athletes. Athletes drink baking soda dissolved in water (0.3 g/kg body weight) 1 h before exercising to neutralise lactic acid produced during physical activity. The research included 200 arrested deliveries (100 patients were given bicarbonate 1 h before oxytocin augmentation). As a result, a significant decrease in AFL concentration with a mean value 0.7 mmol/l (p?0.05) and 1.0 mmol/l (p ? 0.001) was observed in the group with a low and a high (?10.1 mmol/l) AFL level, respectively, in contrast with the group which was not given bicarbonate, where the value of AFL increased significantly to a mean value 1.2 mmol/l after 1 h of stimulation with oxytocin (*p* ? 0.001). The authors conclude that bicarbonate treatment increases the rate of vaginal deliveries in failure to progress in labour.

To verify the actual use of bicarbonate treatment during labour, it is necessary to compare its results with oxytocin augmentation and collect data on potential signs of intolerance, which were not observed in this group of patients.

## Conclusions

The positive predictive value of AFL levels for dysfunctional labour suggests that this measurement can be used as a meaningful tool in the obstetric ward. A considerable number of studies conducted so far indicate that over 90% of labours with a low AFL concentration will be concluded with spontaneous vaginal delivery. It represents a significant step forward in reducing avoidable caesarean sections in this group of patients through individual care and the optimal timing of oxytocin administration. Overall, when it comes to bicarbonate treatment, the literature is limited and requires further research. Considering that a high AFL level is an independent risk factor of a caesarean section due to labour arrest, it is thus possible to avoid prolonged labour, simplify the decision-making process for surgical deliveries and avoid complications in the postpartum period associated with prolonged labour. Measuring the AFL level might simplify the patient’s allocation to a group that will benefit from the administration of oxytocin and to the group that will not benefit from further prolongation of labour, thus requiring a caesarean section.
